# Assessment of effectiveness of health education bundle to overcome vaccine hesitancy in mothers: single blinded randomized study

**DOI:** 10.1186/s13104-025-07291-3

**Published:** 2025-05-23

**Authors:** Mounika Bazar, Kiran N. Baliga, Anupama Nayak Panakaje, S. R. Ravikiran

**Affiliations:** 1 Department of Pediatrics, Kasturba Medical College Mangalore, Manipal Academy of Higher Education, Manipal, India; 2 Department of Pediatric and Preventive Dentistry, Manipal College of Dental Science Mangalore, Manipal Academy of Higher Education, Manipal, India

**Keywords:** Vaccine hesitancy, Vaccine delay, Vaccine preventable diseases

## Abstract

**Objective:**

Vaccine Hesitancy (VH) challenges previously perceived attitudes of a simple dichotomy of “accept” or “reject”. This is not just due to people being uninformed or misinformed but rather due to multiple forms of distrust. It is criticized as a vague notion with an uncertain hypothetical background. Therefore, this study aimed to assess the baseline knowledge and immunization practices of postnatal mothers and their hesitancy after intervention with the health education tool ‘SuBaDRa’ and compare it with those of the control group.

**Result:**

This hospital-based, single-blinded randomized control study was performed for 2 years with *272 (136 per group)* postnatal mothers (booked cases with immunization cards and access to a smartphone) in Karnataka, India. 'SuBaDRa', a tailored health education tool, was used to counsel the intervention group: Presenting **Su**stainable initiatives by the government; assessing mothers' **Ba**seline immunization knowledge and postintervention revaluations via the 'WHO SAGE questionnaire’, **D**issipating knowledge via health education and **R**einforcement with social media **a**pplications. The control group was counseled about essential newborn care. The vaccine delay of infants at birth and at 6, 10, and 14 weeks and 9 months of age was assessed. The secondary outcome measures mothers' attitudes toward immunization postintervention. The vaccine was considered delayed if it was received later than 2 weeks after the recommended age. The characteristics of the study subjects, i.e., postnatal mothers with eligible newborns, were analyzed via descriptive statistics. These characteristics were compared between the intervention and control groups via the Chi-square (χ^2^) test and Fischer’s exact test. The results revealed that the intervention group vaccine delays at 6, 10, 14 weeks and 9 months were 5.9%, 3%, 0.7% and 11.9%, respectively, and the control group vaccine delays were 20%, 28.9%, 55.6% and 48.1%, respectively, with p values of 0.001 at 6 weeks and 0 for the rest, all of which were highly significant.

*Trial registration* The study was registered on Clinical Trials Registry – India (CTRI) with the registration number (CTRI/2021/08/035749), registered on (18/08/2021).

## Keypoints

– What is already known?
- Educational interventions help with vaccine hesitancy.
– What does this study add?
- The introduction of an efficient, stepwise, multidimensional tool for counseling and reinforcement outperformed other approaches, combating vaccine hesitancy.

## Introduction

Throughout history, vaccines have efficiently triggered immune responses against disease-causing pathogens, protecting us from severe illness and reducing the need for hospitalization [1]. Various global immunization programs have nearly/completely eradicated many grievous and potentially deadly diseases. With herd immunity, immunocompromised/allergic individuals have also gained substantial protection against all vaccine-preventable diseases (VPDs). This impact has helped relieve the stress on families by providing greater confidence in children participating in childcare, school, and other activities, ultimately contributing to the rise of the economy. Thus, immunization has been a global health success story, saving millions of lives annually [2].

Vaccines undergo multiple trials in phases per standard protocols and are issued a license only after rigorous testing, establishing their efficacy and safety [3]. Sometimes, they cause mild, predicted side effects (such as local reactions, pain, irritability and fever) that do not last long. Very rarely, they cause an exaggerated allergic reaction (anaphylaxis) within minutes. Unfortunately, too often, we come across people who suffer from illness/disability caused by diseases or families who mourn the devastating loss of a loved one from a condition that could have been prevented through vaccines.

The World Health Organization (WHO) has listed vaccine hesitancy as one of the greatest threats to global health. The Strategic Advisory Group of Experts (SAGE), charged with advising the WHO on immunization, defines vaccine hesitancy as “the delay in acceptance or refusal of vaccines despite availability of vaccination services” [4, 5]. Vaccine hesitancy is complex and context specific, varying across time, place and vaccine. It includes factors such as complacency, convenience and confidence.” [6] Even though the country's vaccination rate is high (93.5% for FY 2023–24), vaccine hesitancy among parents—especially regarding newborn vaccinations—remains a problem in India, where studies show a frequency of approximately 21.1%. (Update on Immunization of Children, Ministry of Health and Family Welfare, Government of India) [7].

An increasing number of strategies are being proposed to encourage parents to accept vaccines. One tactic is to use messaging that is specific to a person's position on the vaccination hesitancy spectrum. For example, asking permission to talk about vaccines should be the first step in a conversation with a parent who has previously denied them. This should be followed by enabling the parent's particular concerns to be explored and asking what would spur a change in stance. [8, 9] Also the study’s findings by Slam T et al., showed that the primary significant predictors of full vaccination coverage are maternal health care services, child size at birth, household wealth status, and maternal educational attainment [10].

In this study, we aimed to assess the effectiveness of a health education bundle, SuBaDRa, a multicomponent tool that we meticulously tailored to include both in-person and telephone motivational methods to overcome vaccine hesitancy in mothers. Reinforcement was performed periodically before due dates to counter parental forgetfulness [11].

## Methods

This was a hospital-based, single-blinded randomized control study performed between September 2020 and September 2022 and was conducted in the postnatal ward of the district’s government maternity hospital, which is in South India. The study was initiated after approval was obtained from the medical school's Institutional Ethical Committee (IEC) and registered in the Clinical Trials Registry–India (CTRI) with the registration number (CTRI/2021/08/035749), registered on 18/08/2021 with the Clinical Trial Registry ({1}). The authors followed the international guidelines mentioned in the Declaration of Helsinki.

The study group was selected via convenience sampling and block randomization. A selected group of mothers on postnatal days 2–3 (booked cases with immunization cards and access to a smartphone) were briefed about the study being conducted. Mothers whose babies were born premature, had congenital anomalies or who required prolonged NICU stays (> 4 weeks) were excluded from the study. Subjects who agreed to participate in the survey were allocated to groups A and B based on lottery picks.

Mothers were first asked to fill out the ‘WHO SAGE questionnaire’ to assess their vaccination status, baseline knowledge and belief about vaccines. Group A (intervention group) was counseled about the benefits of vaccination, using our health education bundle ‘SuBaDRa’, whereas group B (control group) was counseled about essential newborn care and did not receive any information through social media.

**Table:** 

SuBaDRa	
Su	Sustainable initiatives for vaccination by the Government of India (PowerPoint presentation)-highlighting the active vaccination facilities in the country
Ba	Baseline assessment and gap analysis using WHO questionnaire- translated to local language, pretested, validated and then used for the study (WHO SAGE version 1.0)
D	Dissipate knowledge (PowerPoint presentation and group discussion/talk) -PowerPoint presentation explaining the benefits/advantages of considering on-time vaccination -Groups of 8-10mothers were educated, and their queries were answered (duration-20 min)
Ra	Reinforcement via social media applications (such as WhatsApp)- Sending over videos with messages from socially prominent people, promoting the benefits of immunization

After the sessions mentioned above, the healthcare worker appointed to manage and sort out details was introduced to all the mothers, requesting their response to her telephone inquiries. Mothers were routinely discharged as per the obstetrician’s advice.

For reinforcement of immediate-postpartum counseling, videos (promoting vaccination) were sent to mothers in the intervention group via social media applications (such as WhatsApp) one week before vaccination due dates of 5, 9, and 13 weeks and before nine months, as per the National Immunization Schedule (NIS). In our study, the vaccine was considered delayed if it was received later than two weeks after the recommended age [12, 13]. All the information collected by the healthcare worker was screened randomly throughout the study period to maintain efficacy. After the final analysis, mothers were asked to answer the ‘WHO SAGE questionnaire’ orally again, taking inputs over the telephone. The primary outcome measures were the vaccine delay of infants at 6, 10 and 14 weeks and nine months of age. The secondary measure was the attitudes of mothers regarding immunization after the intervention. Immunization data were obtained from the primary health centers through the Accredited Social Health Activist (ASHA).

Determination of immunization status: Vaccination status was determined at 8, 12 and 16 weeks (interim analyses) and a couple of weeks after nine months of age (final analysis) to allow a reasonable time to receive the recommended vaccines at 6, 10 and 14 weeks and nine months, as included in the NIS. Follow-ups were conducted over the telephone, with updates from the local area primary health center’s ASHA. The child was considered to have completed vaccination if he/she received all the recommended vaccines before one year of age.

Assignment method and sample size: The main outcome measures were computed for both the intervention and control groups. Independent variables such as mother’s age, parity, sex, birth weight, impact of COVID-19, > 2 doses taken (government/private clinic) and the APL/BPL card for financial status were also used to assess the comparability of groups and to control for potential confounding factors. To identify a statistically significant improvement of 5% in vaccine acceptance and a power of 90%, a total of 272 mothers—136 per group—were recruited from ~ 7000 annual births that occur annually at this hospital.

Data analysis: Characteristics of study subjects, namely, postnatal mothers with eligible newborns, were analyzed via descriptive statistics such as p values and confidence intervals (significant at < 0.05% and 95%, respectively). These characteristics were compared between the intervention and control groups via the Chi-square (χ^2^) test and Fischer’s exact test. Statistical analyses were performed via SPSS software version 25.0, ARMONK, NY.

## Results

**Fig. 1:**
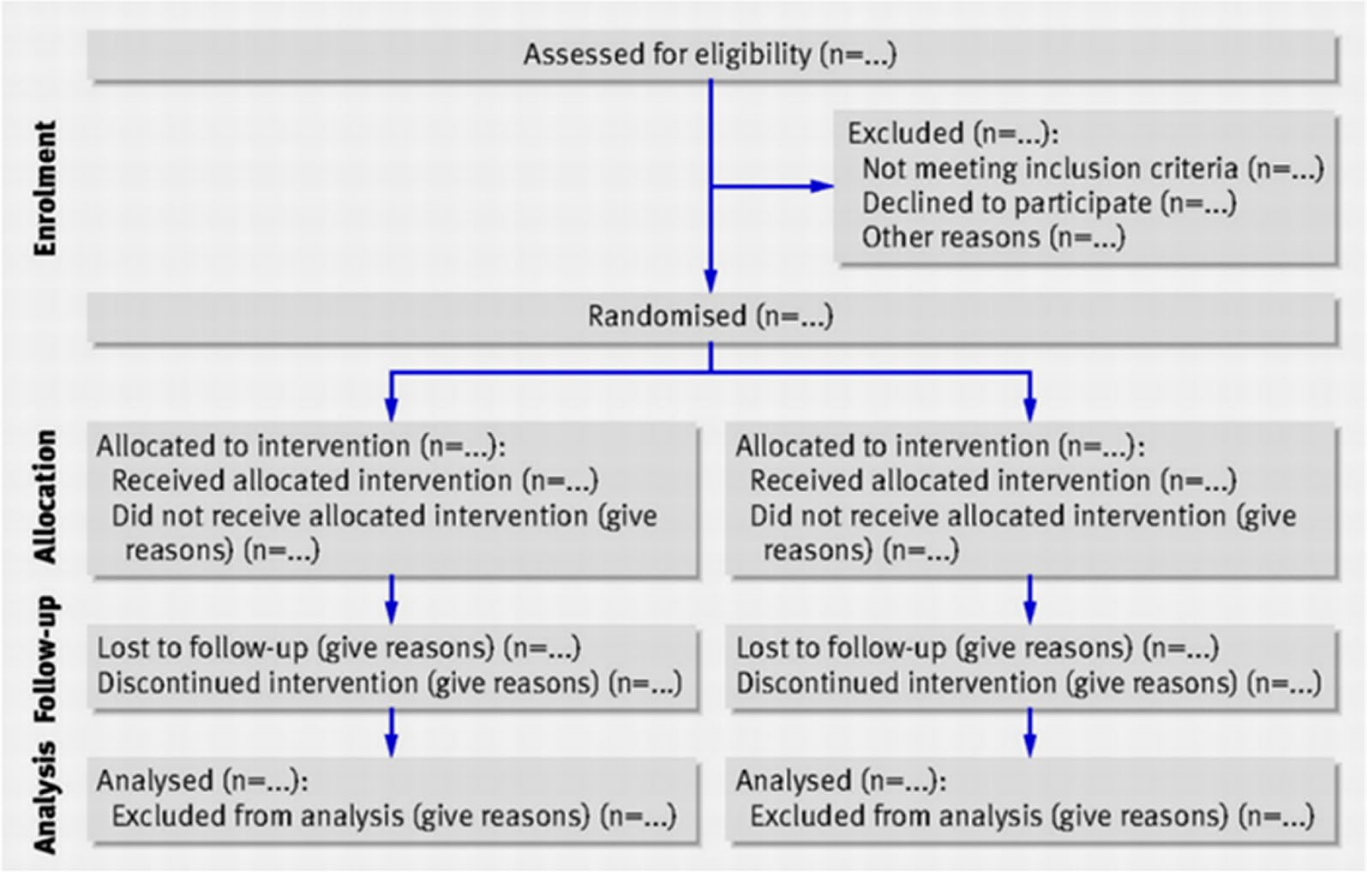
Flow chart of the selection of study subjects in this randomized control trial

Study participants: During our study period, 272 mothers (four pairs of twins) agreed to participate and receive the intervention. Among the corresponding newborns, 136 were assigned to the intervention and control groups. (Fig. 1).

The following sociodemographic categories were considered: mother’s age, parity, sex, birth weight, impact of COVID-19, > 2 doses taken in (government/private clinic) and APL/BPL card for financial status (Table 1). In the intervention group, 49.3% were females and 50.7% were males, whereas in the control group, 46.3% and 53.7% were females and males, respectively. The birth weights of newborns in the intervention group and the ≤ 3 kg group were 57.4% and 42.6%, respectively, and those in the control group were 55.1% and 44.9%, respectively. A total of 80.9% of the participants in the intervention group had no impact on COVID-19, and only 19.1% had an impact, whereas in the control group, only 23.5% had no impact, and 76.5% had an impact on COVID-19 (Table 1).

**Table 1:**
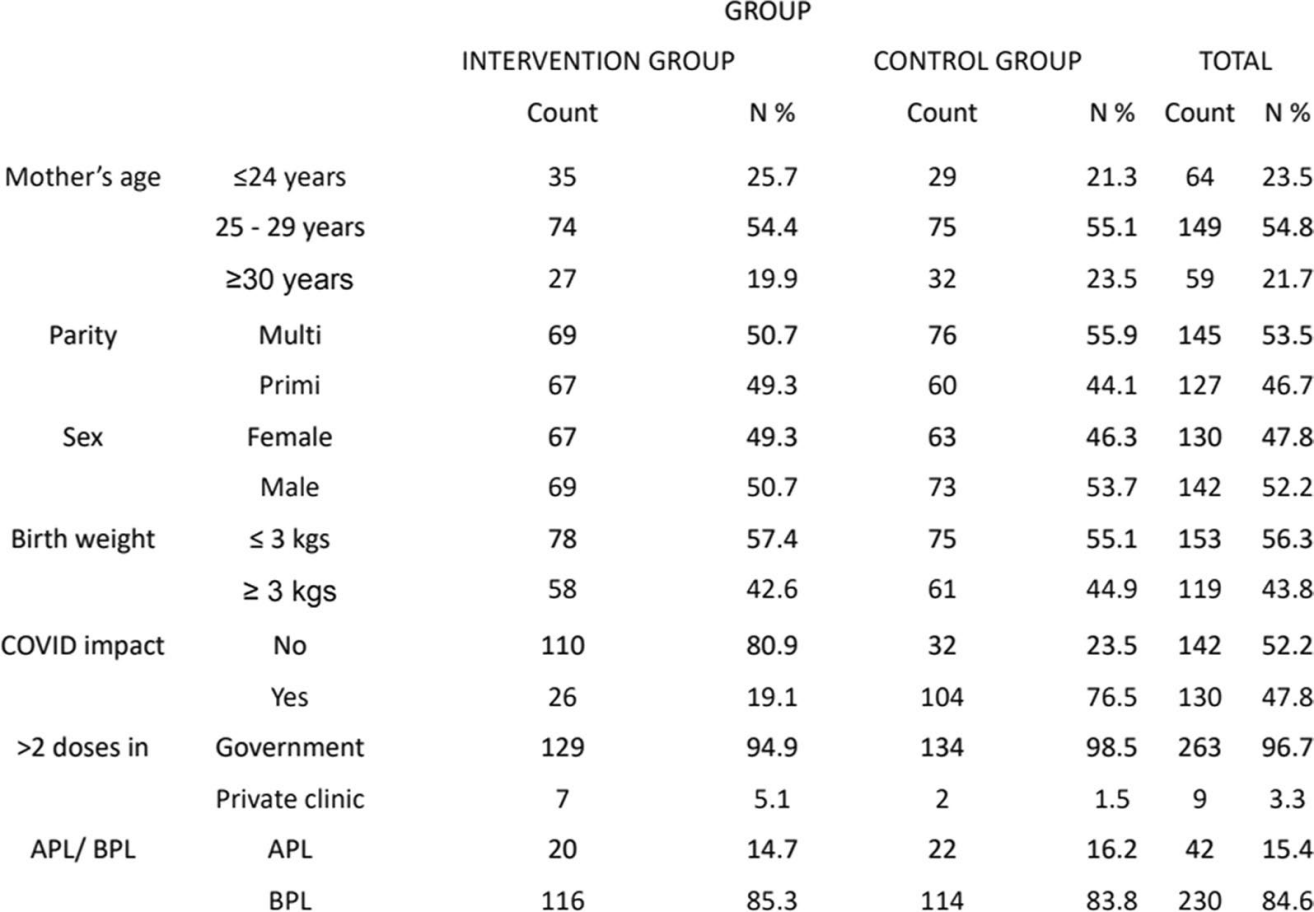
Sociodemographic details of the participating postnatal mothers with their babies

Feasibility of the intervention: Intervention was initiated for 272 subjects. The feasibility of the intervention was 99.2% since 2 mothers had not reported it, 1 from the intervention group and 1 from the control group.

**Fig. 2:**
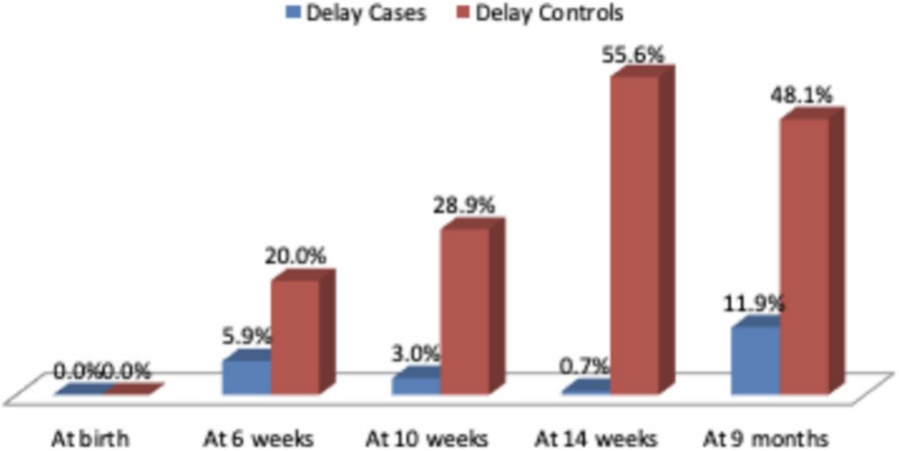
Summary of vaccine delay at various follow-ups

Intervention’s impact on the VA of infants: Vaccine acceptance was significantly greater in the intervention group than in the control group at 6, 10 and 14 weeks and 9 months. At 6 weeks, a 5.9% delay and 94.1% delay were observed in the intervention group, whereas in the control group, a 20% delay and 80% delay were not observed. At 10 weeks, the intervention group presented 3% delay and 97% no delay, whereas the control group presented 28.9% delay and 71.1% no delay. At 14 weeks, the intervention group presented 0.7% delay and 99.3% no delay, whereas the control group presented 55.6% delay and 44.4% no delay. At 9 months, the intervention group had 11.9% delay and 88.1% no delay, whereas the control group had 48.1% delay and 51.9% no delay. All the results were statistically highly significant, with a p value of 0.001 at 6 weeks and 0 for the remaining follow-ups. (Table 2, Fig. 2).

**Table 2:**
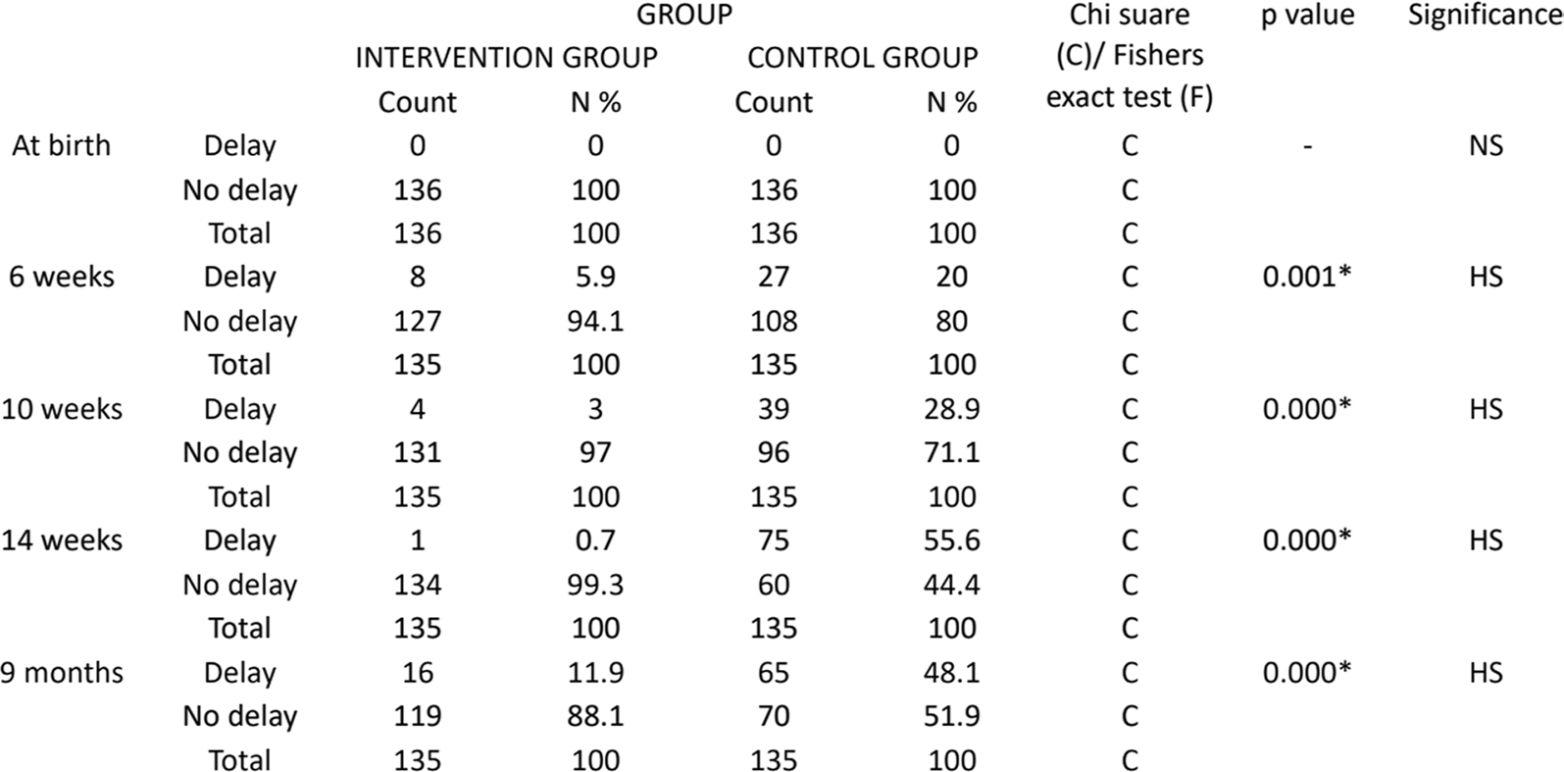
Trend of vaccine delay in the study groups (NS, nonsignificant; HS, highly significant)

Sub analysis was performed for the following independent categories: mother’s age, parity, sex, birth weight, impact of COVID-19, > 2 doses taken in (government/private clinic) and APL/BPL card for financial status. The impact of COVID-19 was significant, with a p value of 0, indicating that this had affected financial status (Table 3).

**Table 3:**
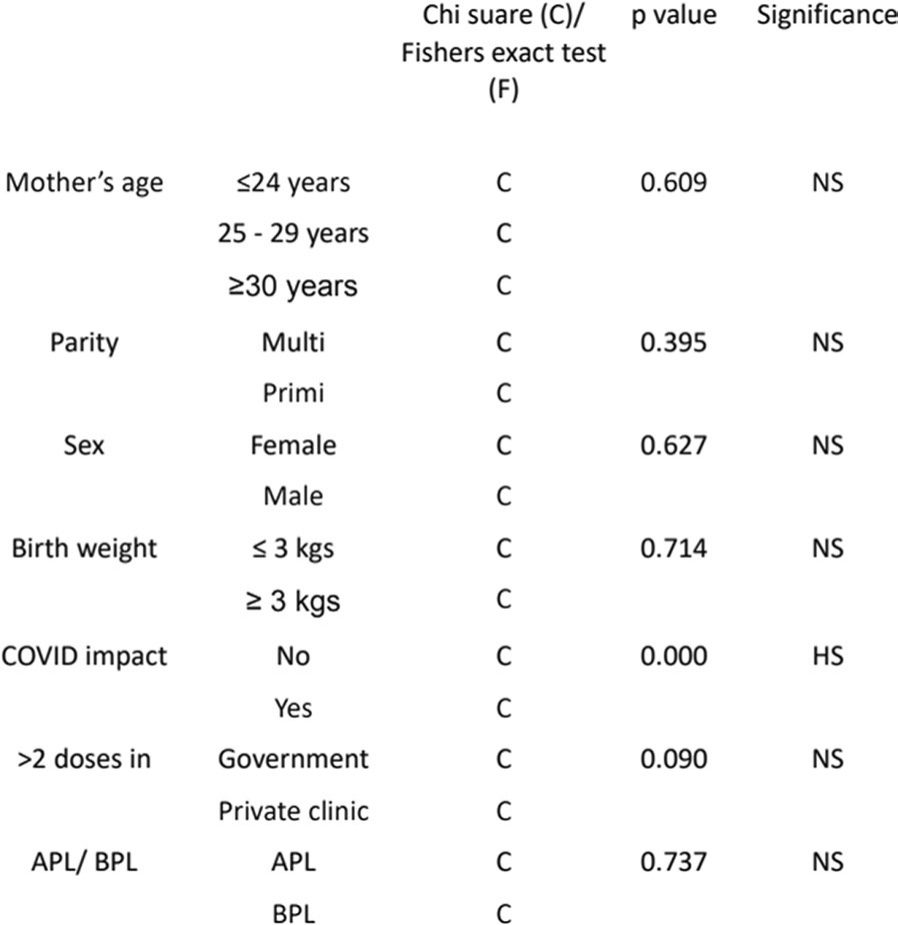
Independent variables of the groups compared (NS – not significant; HS – highly significant)

During motivational counseling, mothers were told about precautions in the vaccination centers to avoid transmission of SARS-CoV-2, such as social distancing, mandatory face masks and frequent sanitation of equipment. The prevalence of COVID-19 as a variable was highly significant in the intervention group.

## Discussion

Immunization has been one of the most successful public health interventions. Immunizing a child requires fool-proof assurance, leading to faith without reservation [14]. Despite many unreliable influences and information in the community, vaccine acceptance (VA) is still remarkably high and stable.

The reasons behind parental vaccine hesitancy or delayed vaccine acceptance are complex and encompass more than just a knowledge deficit [15]. To increase immunization schedule adherence, motivational interviewing [14] and stepwise education [16] have shown significant results. Although mobile apps make it easier to deliver vaccination schedules, their disadvantages—such as trouble with accessibility, the possibility of misinformation, decreased provider engagement, alert fatigue, and data security concerns—may make people more reluctant to receive vaccines. In regard to guaranteeing vaccine adherence and resolving parental concerns, traditional approaches that entail direct communication between healthcare providers continue to be more successful [17].

However, disparate in-person and online intervention forms and the reinforcement of promotions just before these dates have not been attempted. Our health education tool is the first to be developed with an unprecedented increase in significance, confirming previous results. A multifaceted strategy combining behavioral and social determinant tools is needed to address vaccine hesitancy in new parents. Healthcare practitioners and legislators can create successful interventions to increase vaccine confidence by comprehending psychological, social, and systemic obstacles [18]. Vaccine hesitancy, acceptance, and anti-vaccination trends and future prospects for public health. Our study revealed that COVID-19 had a significant effect on financial status since most of the recruited patients were from low socioeconomic groups and were APL/ BPL card holders.

Parents who accept their child’s vaccination can still be anxious and hesitant. They choose to vaccinate only because of the requirements for school entry or because everybody does, and it seems imperative to do so [19]. Vaccine-hesitant individuals constitute a significant fraction of the population and are more attentive than vaccine refusers. Reasons include (a) lack of confidence in the effectiveness and safety of vaccines and vaccine manufacturers and providers [20]; (b) Lack of convenience in terms of the availability and accessibility of vaccine information and facilities in vaccination centers; and (c) Lack of complacency due to the perceived low risk of near eradicating/eradicating VPDs [15]. Ambivalent parents also attribute this to “omission bias” [6], where avoiding adverse reactions (not vaccinating) is considered more acceptable than harm resulting from accepting (vaccinating) [21, 22]. Despite all efforts, sometimes, vaccine refusers do not show up, cancel/miss their appointments, and following them up affects workload and scheduling [5].

Vaccine decision-making is an evolving process. In Uttar Pradesh, a state in Northern India, the community demanded the introduction of the Japanese encephalitis vaccine on the national schedule, providing public access to curb annual disease outbreaks associated with high morbidity and mortality among their children [23]. On the other hand, antivaccine stories often mislead people through social media [24]. Apprehensions such as MMR-induced autism, DTP-induced encephalopathy sudden infant death syndrome (SIDS) and thimerosal-induced neurotoxicity and mercury poisoning contribute to anxiety [25]. Mixed information from educational materials, health journals, web-based resources, and provider messages creates unsatisfactory diversity compared with balanced information and thus requires refinement [26]. Few parents had raised queries such as why the influenza vaccine was suddenly introduced to adolescents when it had not been a part of the schedule earlier. A few other parents are concerned about the cumulative pain and discomfort caused by multiple shots during a single clinic visit. They also wonder if the body can handle multiple antigens introduced at once. Hence, they look for alternative schedules [24, 25, 27].

The most trusted source of information for any parent is their family physician/pediatrician, who can play a crucial role in driving them toward VA, initiating a conversation when they decide to conceive during their prenatal and initial postnatal visits. It is always good to have one’s own vaccination story illustrating an experience in which vaccines become essential just so they feel secure about incidents in general – storytelling with science work wonders. [28]. While their view is being interrogated, benefits must be explained while their concerns are being addressed, as they are transparent about the adverse effects to build trust and address their situation [15, 21]. There exists a fine line between warning people who risk potentially being problematic without factually frightening people. A few mothers were disturbed when they were shown pictures of diseased children, whereas others were keen to see what could happen if they missed their child’s vaccines [29]. It is better to avoid coaxing vaccination sceptics since attempts can sometimes backfire [26, 30].

Immunization is the primary health care provider’s best investment money. The steps required by the government to maintain high uptake of vaccines include minimizing personal and structural barriers such as transport and money, using tailored communication strategies and recruiting health professionals for vaccine education [31].

A similar study revealed that motivational interviewing during the postpartum period increased vaccine acceptance. Another randomized control trial demonstrated that stepwise education beginning at the prenatal period until one month postpartum improved immunization schedule adherence.

## Limitations

Our study aimed to assess the effectiveness of a health education tool, SuBaDRa, in dealing with vaccine acceptors, refusers and those who are hesitant. Although this was a successful intervention, several limitations exist. Initiation of intervention during the antenatal period or earlier would help target this category since most mothers start evaluating the pros and cons of immunization even before they bear the baby or during pregnancy [32]. Most mothers delivering babies at home usually follow primitive traditional practices (including vaccine refusal). Hence, generalizing a community’s status on the basis of the response from booked cases will be erroneous. Since our study was based in a government hospital, most of the study population belonged to the lower socioeconomic class. While hesitancy exists in all population strata, it is often associated with highly educated parents, making it another drawback of this study therefore future studies to be done including more samples from different socioeconomic backgrounds. Despite the flaws mentioned above, the results were still effective. Using public health interventions with a global perspective to target subjects is thus rewarding [33]. Such a significant decrease in vaccine refusal also assures us about other vaccines through childhood and adolescence.

## Conclusion

Health education bundle SuBaDRa, a multidimensional tool to overcome vaccine hesitancy, is the first to demonstrate the effect of stepwise in-person and telephone motivational methods along with reinforcement, with variants in each category and represent one of the most reassuring approaches to vaccine promotion strategies. In addition to the increase in VA, mothers’ knowledge and attitudes toward immunization have significantly improved.

## Data Availability

The datasets used and/or analyzed during the current study are available from the corresponding author on reasonable request.

## Abbreviations

VA: Vaccine acceptance
VH: Vaccine hesitancy
VPDs: Vaccine-preventable diseases
WHO: World health organization
SAGE: Strategic advisory group of experts
NIS: National immunization schedule
SIDS: Sudden infant death syndrome
ASHA: Accredited social health activist
